# Dynamics of NK cell subsets following autologous hematopoietic stem cell transplantation in adult oncologic patients

**DOI:** 10.3389/fimmu.2025.1629118

**Published:** 2025-10-07

**Authors:** Gabirel Astarloa-Pando, Victor Sandá, Ainhoa Amarilla-Irusta, Ainara Lopez-Pardo, Itxaso San Juan, Ainhoa Iturbe-Larrondo, Raquel Pérez-Garay, Silvia Pérez-Fernández, Borja Santos-Zorrozúa, Bárbara Manzanares-Martín, Raquel Bernardo, Carmen González, Alasne Uranga, Mercedes Rey, Marta Alonso, Elena Amutio, Juan J. Mateos-Mazón, Juan C. García-Ruiz, Olatz Zenarruzabeitia, Laura Amo, Francisco Borrego

**Affiliations:** ^1^ Immunopathology Group, Biobizkaia Health Research Institute, Barakaldo, Spain; ^2^ Clinical Analysis Service, Cruces University Hospital, Barakaldo, Spain; ^3^ Scientific Coordination Facility, Biobizkaia Health Research Institute, Barakaldo, Spain; ^4^ Clinical Management Unit of Immunology and Allergy, Reina Sofia Hospital, Córdoba, Spain; ^5^ Maimónides Biomedical Research Institute of Córdoba (IMIBIC), Córdoba, Spain; ^6^ Biogipuzkoa Health Research Institute, Hematology and Hemotherapy Service, Donostia University Hospital, Donostia-San Sebastián, Spain; ^7^ Biogipuzkoa Health Research Institute, Service of Immunology, Donostia University Hospital, Donostia-San Sebastián, Spain; ^8^ Regulation of the Immune System Group, Biobizkaia Health Research Institute, Immunology Service, Cruces University Hospital, Barakaldo, Spain; ^9^ Hematological Cancer Group, Biobizkaia Health Research Institute, Hematology and Hemotherapy Service, Cruces University Hospital, Barakaldo, Spain; ^10^ Department of Genetics, Physical Anthropology and Animal Physiology, Faculty of Science and Technology, University of the Basque Country, Leioa, Spain; ^11^ Ikerbasque, Basque Foundation for Science, Bilbao, Spain

**Keywords:** NK cells, autologous hematopoietic stem cell transplantation, autoHSCT, LAIR-1, CD9, GDF-15, IL-15, TGF-β

## Abstract

Early immune reconstitution following autologous hematopoietic stem cell transplantation (autoHSCT) is associated with improved outcome in various cancers. Natural killer (NK) cells are the first lymphocyte subset to recover post-autoHSCT and play a crucial role in antitumor immunity. In this study, we have performed an in-depth characterization of NK cells in adult patients with different hematological malignancies. Our results revealed that, immediately after autoHSCT, NK cells transiently acquired a decidual-like phenotype, displayed a more immature and activated state, and exhibited an upregulation of inhibitory receptors and a downregulation of activating receptors. This decidual-like and activated phenotype was characterized by increased expression of CD56, CD9, CD49a, CD151, CD38 and HLA-DR. Additionally, we assessed plasma cytokine levels and identified associations between cytokine concentrations and NK cell phenotypic changes. *In vitro* experiments suggested that these phenotype alterations could modulate NK cell function. Finally, in patients with non-Hodgkin lymphoma (NHL), we observed a correlation between NK cell maturation status and progression-free survival. Collectively, our findings provide valuable insights into NK cell dynamics during immune reconstitution following autoHSCT and may inform of strategies for improving patients’ management.

## Introduction

Natural killer (NK) cells play a central role in cancer defense due to their ability to directly kill target cells through multiple mechanisms (1–4). Human circulating NK cells have been classified into two major subsets with different functionalities based on CD56 and CD16 expression: CD56^bright^CD16^low/−^ (CD56^bright^) and CD56^dim^CD16+ (CD56^dim^) NK cells. CD56^bright^ NK cells produce large quantities of immunomodulatory cytokines and chemokines, with limited cytotoxicity unless they are activated by cytokines. In contrast, CD56^dim^ NK cells are more cytotoxic but secrete lower amounts of cytokines (5–8). NK cells express activating and inhibitory receptors whose integrated signals determine their response (9). In this manner, inhibitory receptors such as killer immunoglobulin-like receptors (KIRs) and CD94/NKG2A recognize self-human leukocyte antigen class I (HLA-I) molecules, preventing NK cell activation. On the other hand, NK cells are activated upon recognition of stress-induced ligands on virus-infected or malignant cells, or via antibody-dependent cell-mediated cytotoxicity (ADCC) (4, 7, 10, 11).

Autologous hematopoietic stem cell transplantation (autoHSCT) is an effective and well-established treatment for various hematological malignancies, including multiple myeloma (MM), Hodgkin lymphoma (HL) and non-Hodgkin lymphoma (NHL) with its different subcategories (12–15). Early immune reconstitution following autoHSCT is associated with improved outcome across several cancer types (16–18). Specifically, day 15 absolute lymphocyte count (ALC-15) of ≥ 500 cells/µl after autoHSCT is a good prognostic indicator in MM and NHL patients (17). A high number of NK cells, the first lymphocyte subset to recover post-transplantation (19–21), is associated with better clinical outcome following autoHSCT (22–24).

Given the potent anti-tumor properties of NK cells and their rapid recovery post-autoHSCT, understanding their biology and reconstitution dynamics in this context is of significant importance. It has been described that following autoHSCT, particularly during early leukocyte recovery, there is a redistribution of NK cells subsets. In MM and lymphoma patients it has been described an increased frequency of immature CD56^bright^NKG2A+ NK cells with high levels of CD57 and KIRs early after transplant (25). Another study in MM has described a similar redistribution, characterized by increased NK cell proliferation and a rise in the frequency of both CD56^bright^ NK cells as well as the most immature population within the CD56^dim^ NK cell subset (CD57-NKG2A+) (26). Normally, NK cell function is recovered early after autoHSCT (25, 26), with transcriptomic analyses revealing significant changes in pathways related to the cell cycle, DNA replication, and the mevalonate pathway (27). Notably, in MM patients undergoing autoHSCT, an expansion of a CD9+ decidual-like NK cell subset has been observed, characterized by high granzyme B and perforin expression levels (27). It has been proposed that this CD9+ NK cell subset expansion could be the attributed to the combination of TGF-β and the high levels of IL-15 observed immediately after autoHSCT (28).

NK cell numbers and subset frequencies have been associated with survival outcome (21, 29). For instance, MM patients with lower frequencies of the highly differentiated NKG2A-CD57+ NK cell subset after autoHSCT showed better clinical outcome (26). Additionally, MM patients achieving long-term complete response after autoHSCT showed increased frequencies of NK cells expressing the inhibitory receptors KIR2DL1 and NKG2A compared with age-matched healthy donors (30). Notably, similar to allogeneic HSCT, a graft-versus-tumor effect mediated by NK cells may occur in autoHSCT, potentially influenced by KIR-HLA-I receptor-ligand mismatch and affinity interactions (31–35). KIR and HLA-I genotypes have also been shown to impact neuroblastoma patients undergoing autoHSCT and anti-GD2 therapy (32, 36).

Currently, few reliable biomarkers exist to predict prognosis in patients undergoing autoHSCT, and understanding immune reconstitution complexity is crucial for identifying them. Therefore, a detailed characterization of NK cells and their correlation with prognosis indicators is needed. In this study, we report significant post-autoHSCT changes in the expression of inhibitory and activating receptors, activation markers and decidual-like markers, as well as in plasma levels of key cytokines involved in NK cell functions. We also identified associations between specific cytokine levels and NK cell phenotypic changes. *In vitro* functional studies suggest that these phenotypic alterations may tune NK cell function. Finally, we observed a correlation between NK cell maturation levels and progression-free survival in NHL patients. Overall, these findings provide valuable insight into the NK cell pool reconstitution dynamics after autoHSCT and may contribute to the development of improved therapeutic strategies for cancer patients undergoing this treatment.

## Materials and methods

### Patients´ characteristics and study design

The clinical characteristics of the patients are shown in **Table 1**. Blood samples were collected at six distinct time points: prior to transplantation (S1), post-leucocyte recovery (defined as exceeding 1000 leukocytes/µl of blood, typically around day 12 following autoHSCT) (S2), 30 days post-autoHSCT (S3), 100 days post-autoHSCT (S4), 180 days post-autoHSCT (S5), and one year after autoHSCT (S6) (**Supplementary Figure S1A**). Sample collection was conducted through the Basque Biobank for Research (https://www.biobancovasco.bioef.eus), in accordance with the quality management, traceability, and biosecurity standards outlined in the Spanish Law 14/2007 on Biomedical Research and Royal Decree 1716/2011. The project was approved by the Basque Ethics Committee for Clinical Research (BIO14/TP/003, PI+CES+INC-BIOEF 2017-03). Written informed consent was obtained from all participants in accordance with the Declaration of Helsinki.

**Table 1:** Patients’ clinical characteristics.

		n (%)
Gender	Male	35 (61.4%)
	Female	22 (38.6%)
Cancer type	Hodgkin lymphoma	8 (14.0%)
	Non-Hodgkin lymphoma^a^	41 (72.0%)
	Other cancers^b^	8 (14.0%)
Mobilization regimen	G-CSF	26 (45.6%)
	G-CSF + CTx	17 (29.8%)
	G-CSF + Plerixafor	14 (24.6%)
Conditioning regimen	BEAM Other regimens^c^	40 (70.2%) 17 (29.8%)
Maintenance regimen	Rituximab (Non-Hodgkin lymphoma) Lenalidomide (Multiple myeloma)	5 (8.8%) 2 (3.5%)
	No	50 (87.7%)
Disease progression	Yes	18 (31.6%)
	No	39 (68.4%)
Dead	Yes	8 (14.0%)
	No	49 (86.0%)
		Median (interquartile range)
Age		53 (44-61)
Infused CD34+ cells (x10^6^ cells/kg)		3.3 (2.6-4.2)

a Non-Hodgkin lymphomas are diffuse large B-cell lymphoma (16), follicular lymphoma (11), mantle cell lymphoma (7), angioimmunoblastic lymphoma (3), primary cerebral lymphoma (2) and peripheral T-cell lymphoma (2).

b Other cancers include multiple myeloma (3), acute myeloid leukemia (3), amyloidosis (1) and Burkitt lymphoma (1).

c Other regimens include BUCY (5), Melphalan 200 (4), Thiotepa + BCNU (3), BEA (2), TEAM (2) and Z-BEAM (1).

G-CSF, granulocyte colony-stimulating factor.

BEAM, BCNU, etoposide, cytarabine and melphalan.

BUCY, busulfan, cyclophosphamide.

BEA, BCNU, etoposide, cytarabine.

TEAM, thiotepa, etoposide, cytarabine and melphalan.

Z-BEAM, Zevalin (CD20 targeting antibody) + BEAM.

### Determination of NK cell count

The total number of NK cells was determined from whole blood samples. NK cells were identified as CD45+, CD3-, CD56+ and/or CD16+ by flow cytometry. The following clinical-grade fluorochrome-conjugated monoclonal antibodies (mAbs) were used: FITC anti-CD16 (CLB/FcGran1), PE anti-CD56 (MY31), PerCP-Cy5.5 anti-CD3 (SK7), and V450 anti-CD45 (2D1), all from BD Biosciences. The total NK cell count per μL of blood was calculated using the formula: (percentage of NK cells in the lymphocyte gate x absolute lymphocyte count)/100. The total lymphocyte count was obtained from the hemogram.

### Plasma and peripheral blood mononuclear cells

Plasma and peripheral blood mononuclear cells (PBMCs) were isolated as previously described (28). Briefly, blood samples were collected in EDTA-containing tubes from adults diagnosed with various hematological malignancies who underwent autoHSCT. Plasma was obtained after centrifugation. PBMCs were enriched through density gradient centrifugation, cryopreserved in heat-inactivated fetal bovine serum (FBS) (GE Healthcare Hyclone) with 10% dimethylsulfoxide (DMSO) (Thermo Scientific), and stored in liquid nitrogen until use.

Cryopreserved PBMCs were thawed in a 37°C water bath and washed twice with RPMI 1640 medium supplemented with L-Glutamine (Lonza). Next, cells were incubated for 1 hour at 37 °C with 5% CO_2_ in R10 medium, which consists of RPMI 1640 with GlutaMAX, 10% FBS, and 1% Penicillin-Streptomycin (P-S), all from Thermo Fisher Scientific, and supplemented with 10U DNase (Roche). Subsequently, cells were washed one time, resuspended in NK cell medium (RPMI 1640 medium with GlutaMAX, 10% FBS, 1% P-S, 1% Sodium Pyruvate and 1% MEM Non-Essential Amino Acids Solution, all from Thermo Fisher Scientific), filtered through 70 µm cell strainers, and counted before being used in flow cytometry studies and/or *in vitro* degranulation assays.

### Flow cytometry and data analysis

NK cells were characterized phenotypically (**Supplementary Table S1**, panels 1-3) and functionally (**Supplementary Table S1**, panel 4) by flow cytometry. After two washes with PBS, PBMCs were initially incubated in 1 mL of a 1/1000 dilution of the LIVE/DEAD Cell Stain Kit (Invitrogen) in PBS, on ice for 30 minutes in the dark. Next, cells were washed twice with PBS supplemented with 2.5% bovine serum albumin (BSA) (Sigma-Aldrich) prior to conducting the staining of surface receptors. To achieve this, cells were placed on ice for 30 minutes in the dark and incubated with the fluorochrome-conjugated mAbs listed in **Supplementary Table S1**. Following the staining, cells were washed once more with PBS supplemented with 2.5% BSA and then resuspended in 200 µL of PBS. Samples were acquired using a LSR Fortessa X-20 flow cytometer (BD Biosciences).

Flow cytometry data were analyzed with FlowJo v10.8.1 software. Both manual and automated analyses were conducted. The plug-ins utilized were: DownSample (1.1), UMAP, and FlowSOM (2.6). Briefly, for the automated analysis, events were initially down sampled from the target gate (NK cells) across all samples using the DownSample plug-in. For every donor, NK cells were down sampled to 100 cells. Subsequently, the down sampled populations were concatenated for the analysis. FlowSOM was executed with the specified parameters in every figure.

### NK cell degranulation assay

For *in vitro* NK cell degranulation assays, the P815 mouse mastocytoma cell line was used as the target. Cells were cultured in NK cell medium supplemented with 5 µg/mL of plasmocin (InvivoGen) at 37 °C and 5% CO_2_ in a flask in an appropriate volume. Thawed PBMCs were cultured overnight and the next day they were used in the degranulation assay. Following a previously described protocol (37), PBMCs and P815 cells were co-cultured at an effector-to-target (E:T) ratio of 1:1 (500.000 PBMCs and equal number of P815 cells) per well in round-bottom 96-well plates, in a final volume of 200 µl. Samples from 7 patients at S1, S2 and S3, were used for these experiments. These co-cultures were incubated in the presence of agonist antibodies, previously titrated to determine the optimal concentration in healthy donors. Due to the limited cell numbers in sample S2 and results obtained on healthy donor samples, we selected the anti-LAIR-1 mAb for degranulation assays.

The positive control well contained 0.05 µg/mL of mouse anti-human CD16 (clone 3G8, BD Biosciences, ref. 555404), and 4 µg/mL of mouse IgG1, κ isotype control (clone MOPC-21, BioLegend, ref. 400101). The assay well contained 0.05 µg/mL of mouse anti-human CD16 and 4 µg/mL of mouse anti-human LAIR-1 inhibitory receptor (clone DX26, BD Biosciences, ref. 550810). To test the degranulation, 2 µL of PE-labeled anti-CD107a (clone REA792, Miltenyi Biotec, ref. 130-111-621) were added to each well. After a pulse centrifugation (200g), cells were incubated for 1 hour at 37 °C and 5% CO_2_. Next, 0.66 µL/mL of GolgiStop (BD Biosciences, ref. 554724) and 1 µL/mL of GolgiPlug (BD Biosciences, ref. 550583) were added to each well, followed by another pulse centrifugation and 5 hours of incubation. Finally, plates were stored in the dark at 4 °C overnight, until the next day for antibody staining (**Supplementary Table S1**, panel 4) and subsequent acquisition in a flow cytometer.

### DNA extraction and KIR genotyping

DNA was extracted from PBMCs using the FlexiGen DNA kit (Qiagen) following the manufacturer’s instructions. The initial step involves adding lysis buffer to each sample according to the Qiagen “FlexiGene DNA procedure” flowchart. Briefly, cell nuclei and mitochondria were collected by centrifugation and then resuspended in a denaturation buffer containing QIAGEN Protease. After protein digestion, DNA was precipitated by adding isopropanol, retrieved through centrifugation, washed with 70% ethanol, and dried. DNA was then dissolved in hydration buffer and stored at –20°C for future use.

KIR typing was conducted using a PCR-SSP technique (sequence-specific primers) with the KIR Ready gene kit (Inno-train Diagnostik GmbH) (28). PCR products were amplified, separated on agarose gels, and the results were analyzed following the manufacturer’s guidelines. Depending on the content of KIR genes, the haplotype (A or B) was determined, and genotypes (AA or Bx, with “x” being either A or B) were categorized for every patient. The AA genotype is homozygous for the inhibitory haplotype A (composed of 3DL3, 2DL3, 2DP1, 2DL1, 3DP1, 2DL4, 3DL1, 2DS4 and 3DL2). Haplotype B or Bx genotype includes any combination of KIR other than those mentioned above.

### Plasma determination of cytokines

Plasma cytokine levels were determined in samples that were stored at -80 °C. To measure IL-15 plasma levels, the human IL-15 Quantikine ELISA Kits (R&D Systems) was used following the manufacturer’s guidelines. The optical density was measured in a Varioskan Flash fluorimeter (Thermo Fisher Scientific), and the standard curve along with non-linear regression and log-log line modeling was conducted using GraphPad Prism v.9.3.1 software. To measure TGF-β plasma levels, the Luminex MILLIPLEX TGF-beta 1 Single Plex MAGNETIC Bead Kit (Merck) was used, according to the manufacturer’s guidelines. TGF-β levels were quantified using Luminex^®^ 200™ (Merck) and evaluated with xPONENT^®^ software. To determine GDF-15 plasma levels, Elecsys GDF-15 (Roche) was utilized, following the manufacturer’s guidelines. For quantification, the electrochemiluminescence was measured using a cobas e 801 analytical unit (Roche) immunoassay analyzer.

### Statistical analysis and data representation

For the analysis of panel 1 the sample size was S1 n=44; S2 n=42; S3 n=40; S4 n=38; S5 n=33; S6 n=29. For the analysis of panel 2 the sample size was S1 n=33; S2 n=33; S3 n=32; S4 n=32; S5 n=25; S6 n=22. For the analysis of panel 3 the sample size was S1 n=15; S2 n=15; S3 n=12; S4 n=15; S5 n=10; S6 n=11. Non-parametric Wilcoxon matched-pairs signed-rank test was used to determine significant differences among groups. Correlograms were used to visualize correlations between different variables, with the Pearson correlation coefficient indicated by square size and heat scale. Kaplan-Meier analysis of progression-free survival was performed by dividing the NHL cohort into two groups based on the median value of the variable under analysis. Survival analyses were restricted to the NHL cohort due to statistical power considerations. The limited patient numbers in the Hodgkin Lymphoma and other cancers groups (n=8 each) were insufficient to detect true differences or associations with adequate statistical significance and robustness. The analyses were performed using R (version 4.3.1), a language and environment for statistical computing (R Foundation for Statistical Computing using ggplot2, tidyr and readxl packages). GraphPad Prism v.9.3.1 was also used for graphical representation.

## Results

### Immature and activated NK cells with a decidual-like phenotype expand following autoHSCT

**Figure 1:**
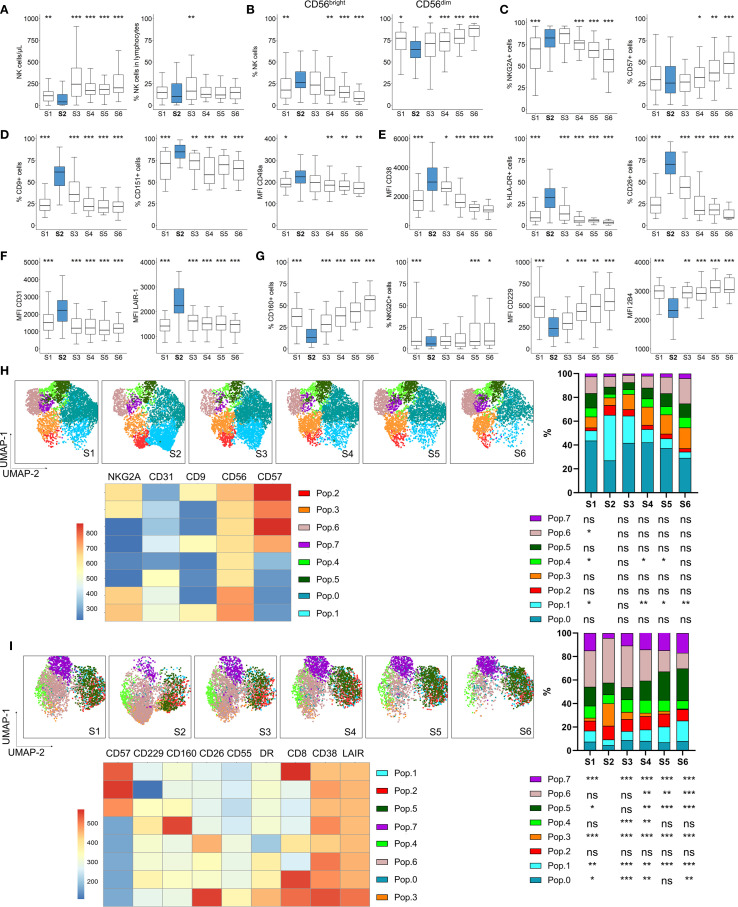
NK cell numbers and phenotype dynamics after autoHSCT. Boxplot graphs illustrating NK cell analysis at six time points: before autoHSCT (S1), after reaching leucocyte recovery (more than 1000 leukocytes/µl, typically around day 12 after autoHSCT) (S2), 30 days (S3), 100 days (S4), 180 days (S5), and one year after autoHSCT (S6). **(A)** Absolute NK cell counts (left) and percentage of NK cells within lymphocytes (right). **(B)** Percentages of CD56^bright^ and CD56^dim^ NK cell subsets. **(C)** Frequencies of NKG2A+ (left) and CD57+ (right) NK cells. **(D)** Percentages of CD9+ (left) and CD151+ (middle) NK cells and median fluorescence intensity (MFI) of CD49a (right). **(E)** MFI of CD38 (left), and frequencies of HLA-DR+ (middle) and CD26+ (right) NK cells. **(F)** MFI of CD31 (left) and LAIR-1 (right) on NK cells. **(G)** Frequencies of CD160+ (left) and NKG2C+ (middle) NK cells and MFI of CD229 (middle) and 2B4 (right). Boxplots display the median and interquartile range (IQR; 25–75th percentiles), with whiskers indicating the minimum and maximum values. Statistical significance was determined by comparing each sample to S2 (blue) using the Wilcoxon matched-pairs signed-rank test: *p < 0.05, **p < 0.01, ***p < 0.001; non-significant values were not indicated. **(H, I)** UMAP projection of CD56+ NK cell populations (Pop) identified by FlowSOM clustering tool for the specified markers from panels 1 **(H)** and 2 **(I)**. Fluorescence intensity of each Pop is indicated in the column-scaled z-score, and bar graphs illustrate Pop distributions across time points (S1-S6). Comparisons were made with S2 using the Wilcoxon matched-pairs signed-rank test (*p < 0.05, **p < 0.01, ***p < 0.001; ns, not significant).

NK cell phenotypic changes occur during immune system reconstitution after autoHSCT (21, 25, 26, 28, 29). In this study, we have analyzed NK cell reconstitution in a cohort of adult patients with hematological malignancies undergoing autoHSCT. Fifty-seven patients were recruited in the Hematology Services of the Cruces University Hospital and the Donostia University Hospital. Peripheral blood samples were collected at multiple time points: before autoHSCT (S1), after reaching leucocyte recovery (>1000 leukocytes/µl, typically around day 12 post-autoHSCT, S2) and 30 days (S3), 100 days (S4), 180 days (S5) and a year (S6) after autoHSCT (**Supplementary Figure S1A**). The clinical characteristics of patients are described in **Table 1**. Following autoHSCT, the absolute number of NK cells significantly declined from S1 to S2, followed by an increase at S3, which was sustained until S6 (**Figure 1A**). However, the proportion of NK cells among total lymphocytes remained unchanged during reconstitution. NK cells were identified based on CD56 and NKp80 expression, while excluding other lineage markers (CD3, CD14, CD19, and CD123), as illustrated in **Supplementary Figure S1B**. We further categorized NK cells into two subsets based on CD56 expression: CD56^bright^ and CD56^dim^. Our analysis revealed a post-autoHSCT increase in the more immature CD56^bright^ NK cell subset and a concomitant decrease in CD56^dim^ NK cells (**Figure 1B**).

Given the observed shift towards an immature NK cell phenotype, we analyzed the expression of the maturation markers NKG2A and CD57. The percentage of NKG2A+ NK cells increased shortly after transplantation (from S1 to S2), while CD57+ NK cells increased at later time-points (>100 days, S4 and S5) (**Figure 1C**). Co-expression analysis of NKG2A and CD57 within NK cell subsets confirmed that the most immature CD56^bright^ NK cells predominantly exhibited a NKG2A+CD57- phenotype. Notably, these NKG2A+CD57- constituted the most frequent subset among total and CD56^dim^ NK cells at all time points (**Supplementary Figure S2**). Their frequency in CD56^dim^ NK cells increased after S1 but gradually declined after S3, while the proportion of terminally differentiated NKG2A-CD57+ NK cells increased.

Previous reports indicate that genes associated with activation and several receptors are differentially expressed in adult MM patients undergoing autoHSCT (27). Among these, the decidual NK cell markers CD9, CD151, and CD49a (38–40) were of particular interest. In this study, we observed a transient increase in NK cells expressing CD9, CD151 and CD49a at S2, returning to pre-transplant levels by S6 (**Figure 1D** and **Supplementary Figure S3A**). Further analysis of CD9 and CD151 expression revealed that the CD9+CD151+ NK cell subset increased fourfold at S2 compared to S1, whereas the CD9- subset decreased proportionally (**Supplementary Figure S3B**), suggesting that CD9 is a predominant marker in this characteristic decidual-like phenotype post-autoHSCT. Since decidual NK cells are characterized by higher CD56 expression (39), we analyzed the MFI of CD56 in populations expressing CD9 and CD151. CD56 MFI increased at S2 (**Supplementary Figure S3C**), with CD9+CD151- NK cells displaying the highest CD56 expression (**Supplementary Figure S3D**). This aligns with previous reports indicating that CD9 is a specific marker of decidual and decidual-like NK cells, whereas CD151 is also expressed on peripheral NK cells (39, 41).

Analyses of activation markers revealed a transient increase in CD38 expression at S2, along with a rise in HLA-DR+ and CD26+ NK cells (**Figure 1E**, **Supplementary Figure S4A**), all of which are indicative of NK cell activation (42–44). Additionally, inhibitory receptors expression levels were upregulated at S2, including CD31, LAIR-1 (**Figure 1F**, **Supplementary Figure S4B**) and NKG2A (**Figure 1C**, a maturation marker, which is also an inhibitory receptor). Conversely, regarding activating receptors, the proportions of CD160+ and NKG2C+ NK cells decreased at S2, as well as the MFI of CD229 and 2B4 (also known as CD244) (**Figure 1G**, **Supplementary Figure S4C**).

Further analysis of additional receptors revealed an increase in CD55 expression, a potential inhibitory receptor and decidual NK cell marker in mice (45, 46) at S2 (**Supplementary Figure S5**). Previous reports have shown that expression of genes encoding for chemokine receptors, such as CCR5 and CCR7, was altered after autoHSCT in MM patients (27). In our study, the expression of CCR5, a homing receptor for infected tissues and tumors (47, 48), was upregulated at S2 (**Supplementary Figure S5**), while other chemokine receptors remained unchanged (data not shown). Interestingly, inhibitory receptor Siglec-7, which is associated with a highly functional NK cell subset (49, 50), exhibited a unique trend, decreasing at S2 (**Supplementary Figure S5**), in contrast to other inhibitory receptors. Activating receptor CD226 (DNAM-1) remained unchanged, whereas CD8, a marker linked to NK cell cytotoxicity (51), increased at S2, primarily in the CD56^bright^ NK cell subset (**Supplementary Figure S5**).

To complement our supervised analysis, we conducted unsupervised clustering using uniform manifold approximation and projection (UMAP) and FlowSOM methodologies (**Figures 1H, I**). Among CD56+ NK cells, the analysis identified 8 distinct NK cell populations (or Pops) based on the expression of 5 (NKG2A, CD31, CD9, CD56, CD57) and 9 (CD57, CD160, CD229, CD55, CD38, LAIR-1, CD8, HLA-DR and CD26) surface markers analyzed in panel 1 and 2, respectively (**Supplementary Table S1**). In panel 1 (**Figure 1H**), we observed a significant increase in Pop 1 at S2, characterized by high expression of NKG2A, CD31, CD9, and CD56, but low CD57 expression. Conversely, Pop 4 and Pop 6 frequencies decreased at S2, with Pop 6 displaying higher CD57 levels. In panel 2 (**Figure 1I**), Pop 3 increased at S2, and is characterized by low CD57, CD229, and CD160 levels but high CD26, CD55, HLA-DR, CD38, and LAIR-1 expression. In contrast, Pop 7, characterized by high CD160 expression, decreased at S2. Additionally, Pop 6 decreased while Pop 5 increased after S4, coinciding with CD57 recovery. These unsupervised analysis confirmed the findings from our supervised approach.

Taken together, our results demonstrate that early after autoHSCT, NK cells acquire a more immature, activated, and decidual-like phenotype, with a receptor repertoire skewed toward an inhibitory profile. This is reflected by the transient increase in the expression of inhibitory receptors and the concurrent decrease in activating receptors.

### Plasma cytokine profile following autoHSCT

**Figure 2:**
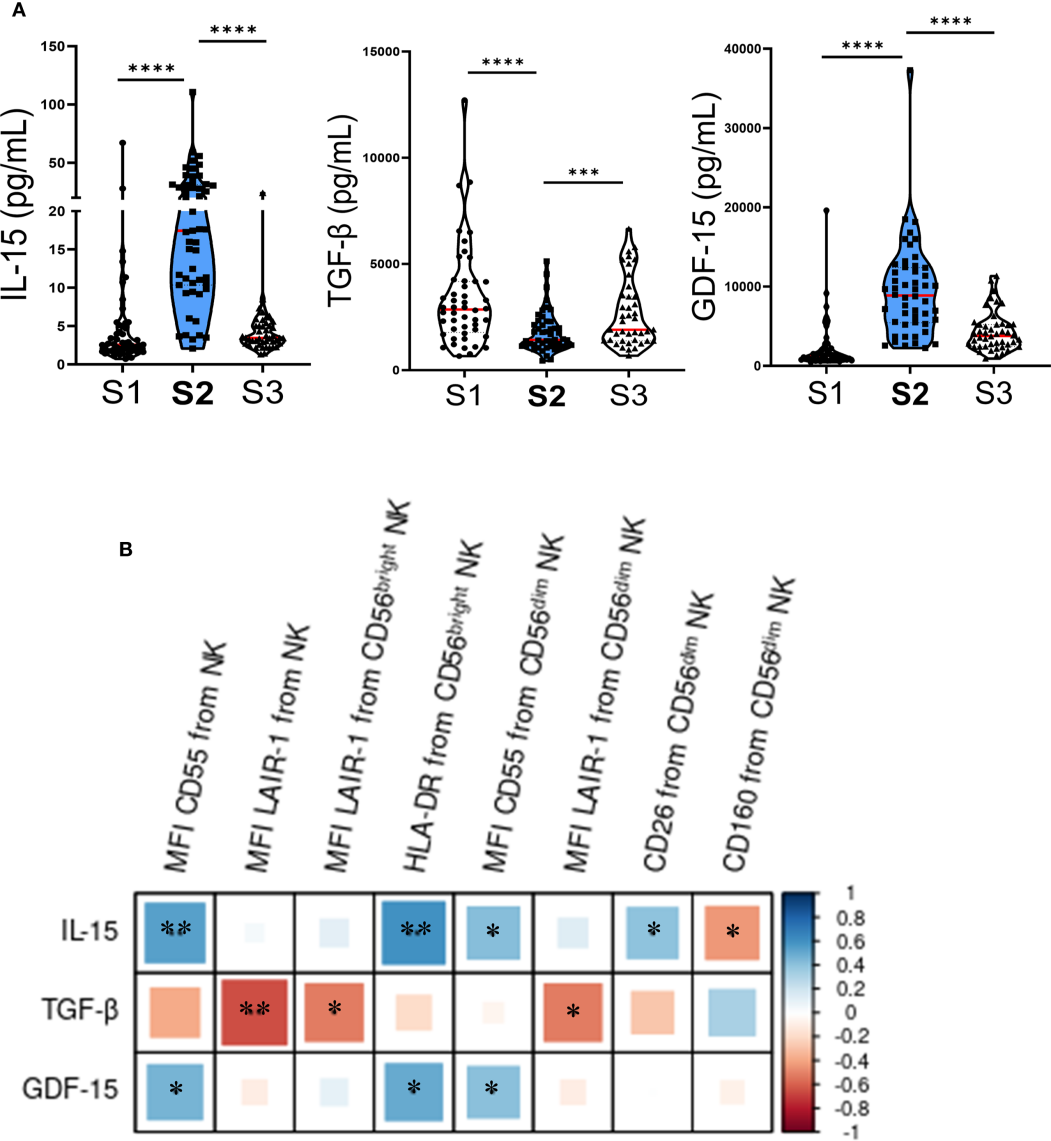
Plasma cytokine levels change early after autoHSCT and correlate with NK cell markers. **(A)** Violin plots depicting IL-15, TGF-β, and GDF-15 plasma levels at S1 (pre-autoHSCT), S2 (leukocyte recovery, around day 12), and S3 (30 days post-autoHSCT). Medians are indicated in red (S1 n=52; S2 n=51; S3 n=44) and IQR (25th–75th percentiles) in green. Comparisons with S2 were performed using the Wilcoxon matched-pairs signed-rank test (***p < 0.001, ****p < 0.0001). **(B)** Correlogram showing Pearson correlation coefficients between flow cytometry markers and cytokine plasma levels at S2. *p < 0.05, **p < 0.01; non-significant values not shown.

We have previously observed that certain cytokines present immediately after autoHSCT may contribute to the acquisition of a decidual-like phenotype by NK cells (28). To investigate the influence of these cytokines on other markers, we quantified their plasma levels and analyzed their correlation with the expression of previously studied receptors in NK cells. Consistent with previous findings in both adult and pediatric patients (24, 26, 28), we observed a significant increase in IL-15 plasma levels shortly after autoHSCT in this cohort (**Figure 2A**). Next, correlation analyses were performed between flow cytometry data and plasma cytokine levels, although in **Figure 2B** we only displayed those correlations that yielded statistically significant results. Furthermore, IL-15 levels at S2 positively correlated with the expression of CD55, HLA-DR and CD26 (**Figure 2B**). Given that these markers are also upregulated at S2, this suggests that elevated IL-15 levels may contribute, at least partially, to the activation phenotype of NK cells at this time point.

In contrast, plasma levels of TGF-β significantly decreased at S2 (**Figure 2A**). Additionally, we observed a negative correlation between plasma levels of TGF-β at S2 and the expression of LAIR-1, as well as a non-significant trend suggesting that TGF-β might counteract the effect of IL-15 on CD55, HLA-DR and CD26 expression (**Figure 2B**). Lastly, we examined the plasma levels of growth differentiation factor 15 (GDF-15) and found a significant increase at S2 (**Figure 2A**). GDF-15 levels positively correlated with the expression of CD55 and HLA-DR (**Figure 2B**), further supporting its potential involvement in shaping the post-transplant NK cell phenotype.

### KIR expression increases following autoHSCT

**Figure 3:**
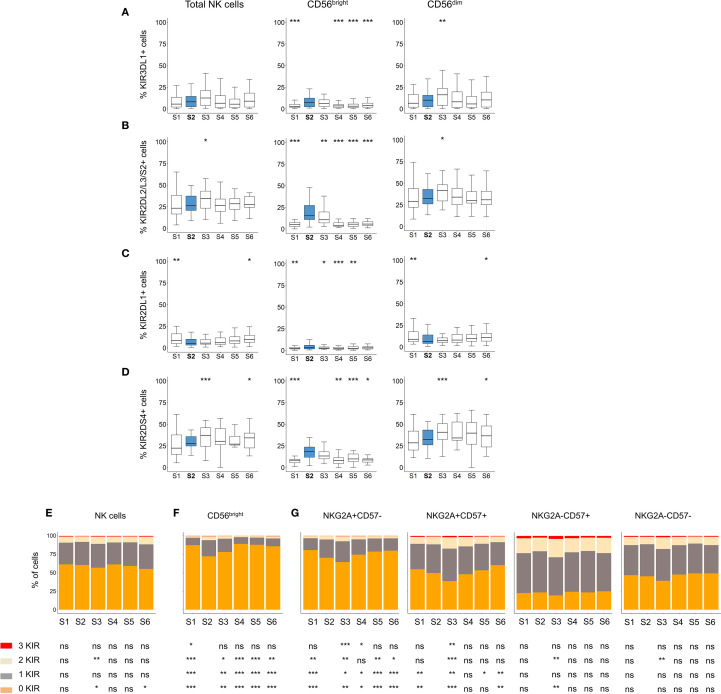
Analysis of KIR expression patterns in NK cells after autoHSCT. **(A-D)** Boxplot graphs showing the frequency of cells expressing KIR3DL1 **(A)**, KIR2DL2/L3/S2 **(B)**, KIR2DL1 **(C)** and KIR2DS4 **(D)** within total NK cells, as well as CD56^dim^ and CD56^bright^ NK cell subsets at S1-S6. Boxplots depict the median, IQR (25th–75th percentiles), and whiskers (minimum and maximum values). Significance was assessed by Wilcoxon matched-pairs signed-rank test relative to S2 (*p < 0.05, **p < 0.01, ***p < 0.001; non-significant values not indicated). **(E-G)** Bar graphs showing the percentage of NK cells expressing 0 (orange), 1 (grey), 2 (light yellow), or 3 (red) KIR (KIR2DL1, KIR2DL2/L3/S2 and KIR3DL1) across time points (S1-S6). KIR combination analyses in total NK cells **(E)**, CD56^bright^ NK cells **(F)**, and in CD56^dim^ NK cells according to NKG2A and CD57 expression **(G)**. Data are represented as the mean of the percentages of each cell subpopulation. Wilcoxon matched-pairs signed-rank test comparisons with S2: *p < 0.05, **p < 0.01, ***p < 0.001; ns, not significant.

KIRs regulate the ability of NK cells to recognize and kill tumor cells. KIR-ligand genotypes have been associated with patient outcomes in neuroblastoma (36), and changes in KIR expression have been observed following HSCT (25, 27). Therefore, we investigated KIR expression in our cohort. First, we analyzed the KIR haplotypes of the patients (**Supplementary Table S2**). We then assessed KIR expression exclusively in patients carrying the genes encoding the analyzed KIRs. Overall, we observed a transient increase in the percentage of KIR3DL1+, KIR2DL2/L3/S2+, KIR2DL1+ and KIR2DS4+ NK cells early after autoHSCT, predominantly within the CD56^bright^ NK cell subset (**Figures 3A-D**). However, no association was found between KIR expression and disease progression (data not shown).

During NK cell differentiation, CD56^dim^ NK cells progressively lose NKG2A expression while acquire CD57 and KIR (52). To further investigate this process, we analyzed the number of expressed KIRs (KIR3DL1, KIR2DL2/L3/S2 and KIR2DL1) across different NK cell subsets. No significant changes in KIR expression were observed on total NK cells after transplantation (**Figure 3E**). However, within the CD56^bright^ subset, we detected a significant increase in the frequency of cells expressing one, two and tree KIRs at S2 (**Figure 3F**). Also, and given the altered maturation status of NK cells after autoHSCT (**Figure 1E**), particularly within the CD56^dim^ subset, we examined KIR expression across different maturation stages. Immature CD56^dim^ NK cells (NKG2A+CD57-) exhibited lower KIR expression compared to mature (NKG2A-CD57+) CD56^dim^ NK cells (**Figure 3G**). Notably, both immature NKG2A+CD57- and NKG2A+CD57+ subsets showed a significant increase in KIR-expressing cells at S2 (**Figure 3G**). These findings suggest that KIR expression is transiently upregulated in immature NK cells early after autoHSCT at S2.

### NK cell phenotype impacts NK cell function and correlates with progression-free survival in NHL patients undergoing autoHSC

To assess the clinical relevance of our findings, we first performed correlation analyses between the expression of markers that significantly changed after autoHSCT and relapse in the largest subcohort of patients with NHL (diffuse large B-cell lymphoma, follicular lymphoma, and mantle cell lymphoma) (**Table 1**) undergoing autoHSCT. However, no significant correlation was observed (data not shown). We then conducted Kaplan-Meier analysis, which revealed that NHL patients with lower frequencies of immature NKG2A+CD57- NK cells or higher frequencies of mature CD57+ NK cell subset at S3 exhibited significantly improved progression-free survival (PFS) (**Supplementary Figures S6A, B**). Additionally, we observed a trend suggesting that patients with lower levels of GDF-15 at S2 tended to have better PFS, although this association did not reach statistical significance (**Supplementary Figure S6C**). Given the clinical heterogeneity and limited sample size of our cohort, these findings require validation in larger, more homogeneous patient populations.

**Figure 4:**
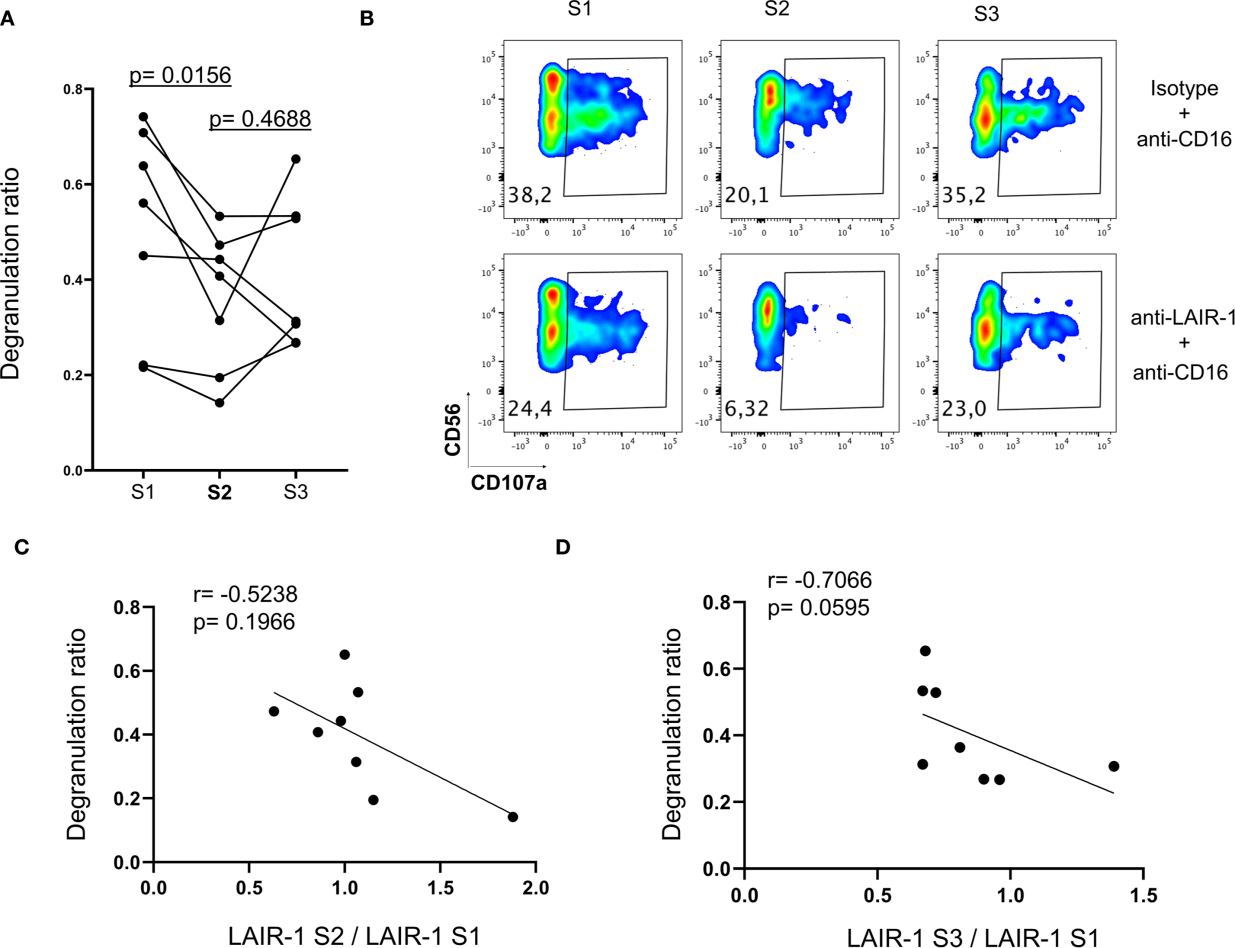
The impact of receptor repertoire changes in NK cell function. NK cells from patients at S1, S2 and S3 were analyzed for degranulation (CD107a) response to antibodies stimulation (see Materials and Methods). **(A)** Degranulation ratio is calculated according to the formula: percentage of CD107a+ NK cells in the presence of anti-LAIR-1 and anti-CD16 antibodies/percentage of CD107a+ NK cells in the presence of isotype control and anti-CD16 antibodies. Each point represents an individual patient (n=7). Comparisons with S2 were using Wilcoxon matched-pairs signed-rank test. **(B)** Representative degranulation (CD107a) data from a patient. **(C)** Correlogram depicting the Spearman correlation between LAIR-1 expression ratio (MFI of LAIR-1 at S2/MFI LAIR-1 at S1) and the degranulation ratio at S2. **(D)** Correlogram showing the Spearman correlation between LAIR-1 expression ratio (MFI of LAIR-1 at S3/MFI LAIR-1 at S1) and degranulation ratio at S3.

Our data indicate that inhibitory receptors such as LAIR-1 are upregulated, while activating receptors downregulated at S2 (**Figure 1**). Based on these findings, we hypothesized that the altered receptor repertoire observed at S2 might affect NK cell function. To test this hypothesis, we performed *in vitro* functional assays using patient-derived PBMCs (S1-S3) and agonist antibodies against LAIR-1 inhibitory receptor in a previously described P815-based degranulation assay (37) (see Materials and Methods). We tested the inhibitory effect of LAIR-1 in CD16-mediated degranulation. At S2, NK cell CD16-mediated degranulation significantly decreased (**Figures 4A, B**), coinciding with increased LAIR-1 expression (**Figure 1F**). Moreover, we observed a negative correlation between LAIR-1 upregulation and the degranulation capacity, although this did not reach statistical significance (**Figures 4C, D**). These findings suggest that increased expression of inhibitory receptors may contribute to impaired NK cell function early after autoHSCT.

## Discussion

In this study, we have investigated the reconstitution of NK cells in adult patients with various hematological malignancies following autoHSCT. We analyzed phenotypic alterations in NK cell subsets, measured plasma cytokine levels relevant to NK cell function, conducted *in vitro* functional assays to explore the impact of these phenotypic changes, and performed data analysis to identify potential prognostic biomarkers and elucidate mechanisms underlying NK cell phenotype modulation early after autoHSCT. Previous studies have reported substantial changes in the NK cell surface phenotype and the transcriptome following autoHSCT (25–28). Our findings reveal a shift towards a decidual-like, immature and activated phenotype, with an increased expression of inhibitory receptors and a reduction in activating receptors early after autoHSCT in lymphoma patients. This activated and decidual-like phenotype is characterized by an upregulated expression of CD56, CD9, CD49a, CD151, CD38, and HLA-DR shortly after autoHSCT. Furthermore, plasma cytokine levels were associated with these phenotypic changes, and the *in vitro* experiments suggest that some of these alterations may modulate NK cell function. Specifically, LAIR-1’s increased expression early after autoHSCT is able to inhibit CD16-mediated degranulation (**Figure 4**). Collagen is the ligand of LAIR-1 (53, 54). Interestingly, the upregulation of collagen expression by tumor cells and/or tumor stroma could lead to the downregulation of anti-tumor responses mediated by LAIR-1 expressed on NK cells (55, 56). This suggests that in certain specific circumstances, the increased expression of LAIR-1 following autoHSCT may have a role in controlling tumor growth. However, we have not seen a correlation between relapse and expression levels of LAIR-1 in our cohort. Undoubtedly, more studies in similar and other cohorts (perhaps in patients with solid tumors undergoing autoHSCT) are required to properly assign a role to LAIR-1 overexpressing NK cells in tumor control following autoHSCT. Regarding 2B4, it has been previously shown to deliver an inhibitory signal in decidual NK cells due to deficient expression of the signaling lymphocyte activation molecule (SLAM)–associated protein (SAP) at both mRNA and protein levels (57). However, in our study, we considered 2B4 an activating receptor because we are examining peripheral blood NK cells, not decidual NK cells. Furthermore, supporting our interpretation, our previous data on peripheral blood NK cells showed no significant change in *SH2D1A*, the gene encoding SAP, transcript levels in the S2 sample compared to S1 and S3 (27). This strongly suggests that, in the context of autoHSCT in peripheral blood, 2B4 primarily functions as an activating receptor.

The cytokine milieu plays a crucial role in shaping the NK cell phenotype in cancer patients undergoing autoHSCT. Correlation analyses (**Figure 2B**) indicated that the increased expression of activation markers such as HLA-DR and CD26, as well as receptors like CD55, could be driven, at least in part, by elevated IL-15 levels at S2 (**Figure 2A**). This aligns with previous studies, highlighting the importance of IL-15 in NK cell activation (28, 43, 58–60). Conversely, TGF-β exhibited a tendency to counteract the effects of IL-15 (**Figure 2B**); however, IL-15 appeared dominant, as marker expression correlated more strongly with IL-15 than TGF-β levels, in agreement with prior findings (28).

High frequencies of circulating CD9+ NK cells have been associated with poor cancer prognosis (61–64), suggesting that CD9 could serve as a prognostic biomarker in cancer patients. While CD9+ NK cells have been linked to prognosis in other cancer settings, our findings, coupled with our previous observations in MM and pediatric oncology patients, suggest that the robust expansion of this subset following autoHSCT is a phenomenon occurring independently of the specific underlying malignancy (27, 28). Furthermore, we believe that our data suggest that NK cell dynamics, at least for the parameters investigated in this article, are primarily influenced by the auto-HSCT procedure rather than by disease-specific heterogeneity, a finding supported by our previous work (26–28). Nevertheless, larger and more homogeneous patient cohorts will be necessary to establish CD9+ NK cells as a definitive prognostic biomarker.

In our cohort of hematological cancer patients, we observed that NK cells with an immature phenotype (NKG2A+CD57-) were most frequent at S2, whereas the more mature subset (NKG2A-CD57+) had the lowest frequencies at this time point, followed by a gradual increase, reaching peak levels at S5 and S6. This aligns with established NK cell developmental models, in which NK cells progressively lose NKG2A and acquire CD57 expression as they mature (29, 52). Immature CD56^bright^ and NKG2A+CD57- NK cells expressing KIR expanded at S2 (**Figure 3**), consistent with previous observations in pediatric oncologic patients (28). These cells may represent NK cells that have upregulated CD56 expression in response to IL-15 stimulation (58).

Identifying novel prognostic biomarkers is of significant clinical relevance. We observed that NHL patients with lower frequencies of immature NKG2A+CD57- NK cells or higher frequencies of mature CD57+ NK cells at day +30 after autoHSCT (S3) exhibited superior PFS (**Supplementary Figures S6A, B**). This contrasts with previous findings in MM patients, where lower frequencies of mature CD57+NKG2A- NK cells were associated with improved clinical outcomes (26). These discrepancies may stem from differences in malignancies (MM vs. lymphomas) and conditioning regimens (melphalan 200 mg/m² vs. BEAM chemotherapy). In lymphomas, NKG2A-expressing NK cells may contribute to poorer prognosis due to potential inhibition by HLA-E expressing tumor cells (65, 66).

Finally, we observed a trend suggesting that NHL patients with lower GDF-15 levels at S2 tended to have improved PFS (**Supplementary Figure S6C**). GDF-15 has been implicated in NK cell inhibition in inflammation, pregnancy, and various cancers (67). Furthermore, serum levels of GDF-15 has been proposed as a biomarker in patients with cancer (68, 69). Although the mechanism of GDF-15 in cancer has long remained elusive, recent studies have demonstrated that it impairs LFA-1-mediated adhesion of T cells to activated endothelial cells, which is a crucial step for T cell extravasation (70). A similar inhibitory effect on NK cell trafficking is plausible; however, further studies are required to confirm this hypothesis.

Despite these findings, our results should be interpreted with caution due to the relatively small and heterogeneous nature of our cohort. Further studies involving larger and more homogenous patient populations will be required to validate these observations and confirm their clinical relevance. Nevertheless, our results provide understanding into the dynamics of the NK cell pool reconstitution following autoHSCT and may help to develop therapeutic strategies for cancer patients undergoing this treatment.

## Data Availability

The raw data supporting the conclusions of this article will be made available by the authors, without undue reservation.
