# Supplementary material for: Dynamics of NK cell subsets following autologous hematopoietic stem cell transplantation in adult oncologic patients
Source: Front Immunol. 2025 Oct 7;16:1629118. doi: 10.3389/fimmu.2025.1629118 (PMC12537779; doi:10.3389/fimmu.2025.1629118)
Supplement: Supplementary file 1 [file DataSheet1.pdf]

Supplemental Information

Dynamics of NK cell subsets following autologous hematopoietic stem cell transplantation in adult oncologic patients

Gabirel Astarloa-Pando, Victor Sandá, Ainhoa Amarilla-Irusta, Ainara Lopez-Pardo, Itxaso San Juan, Ainhoa Iturbe-Larrondo, Raquel Pérez-Garay, Silvia Pérez-Fernández, Borja Santos-Zorrozúa, Bárbara Manzanares-Martín, Raquel Bernardo, Carmen González, Alasne Uranga, Mercedes Rey, Marta Alonso, Elena Amutio, Juan J. Mateos-Mazón, Juan C. García-Ruiz, Olatz Zenarruzabeitia, Laura Amo*, Francisco Borrego*.

*Correspondence: Laura Amo and Francisco Borrego. Immunopathology Group, Biobizkaia Health Research Institute, 48903 Barakaldo, Spain. E-mails: laura.amoherrero@bio-bizkaia.eus; francisco.borregorabasco@bio-bizkaia.eus

This file includes:

Figure S1. Sample acquisition and gating strategy for NK cell identification.

Figure S2. NK cell maturation status after autoHSCT.

Figure S3. Transient acquisition of a decidual-like phenotype by NK cells early after autoHSCT.

Figure S4. NK cell activation markers and expression of inhibitory and activating receptors after autoHSCT.

Figure S5. Additional alterations in NK cell receptors expression following autoHSCT.

Figure S6. Altered NK cell phenotype and its potential impact on patient prognosis.

Table S1. Flow cytometry panels.

Table S2. Patients´ KIR haplotypes.

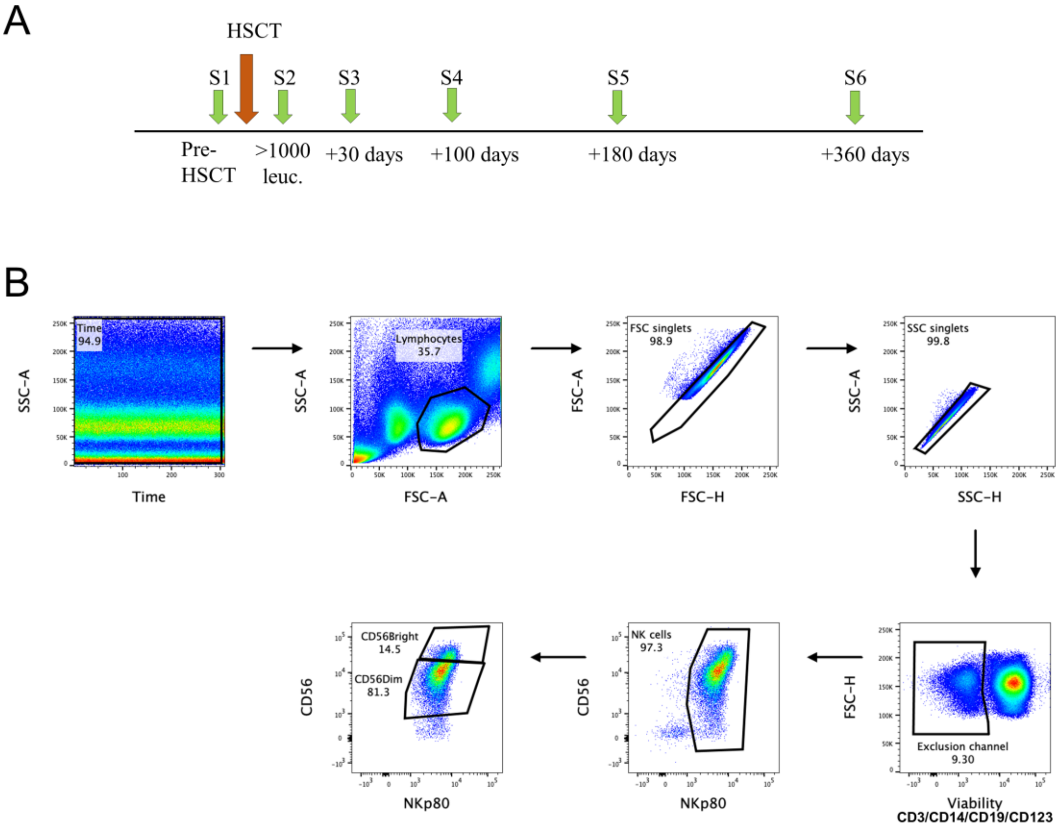

Figure S1. Sample acquisition and gating strategy for NK cell identification. (A) Samples from adult patients with hematological malignancies were collected at six time points: before transplantation (S1), after achieving leucocyte recovery (≥1000 leukocytes/µl, typically around day 12 post-autoHSCT) (S2), and at 30 days (S3), 100 days (S4), 180 days (S5), and one year (S6) post-autoHSCT (S6). **(B)** Representative pseudocolor plots illustrating the gating strategy for identifying total NK cells and NK cell subsets. The analysis workflow include: (i) exclusion of irregular acquisition events using a time gate, (ii) selection of lymphocytes based on forward and side scatter properties, (iii) gating of single cells, and (iv) identification of NK cells by excluding cells positive for the viability marker and lineage markers (CD3, CD14, CD19, and CD123). Total NK cells were further defined as NKp80+ cells within this population. NK cell subsets were further classified into CD56^{bright} and CD56^{dim} populations based on CD56 expression. The expression of various markers was analyzed within total NK cells and their subsets.

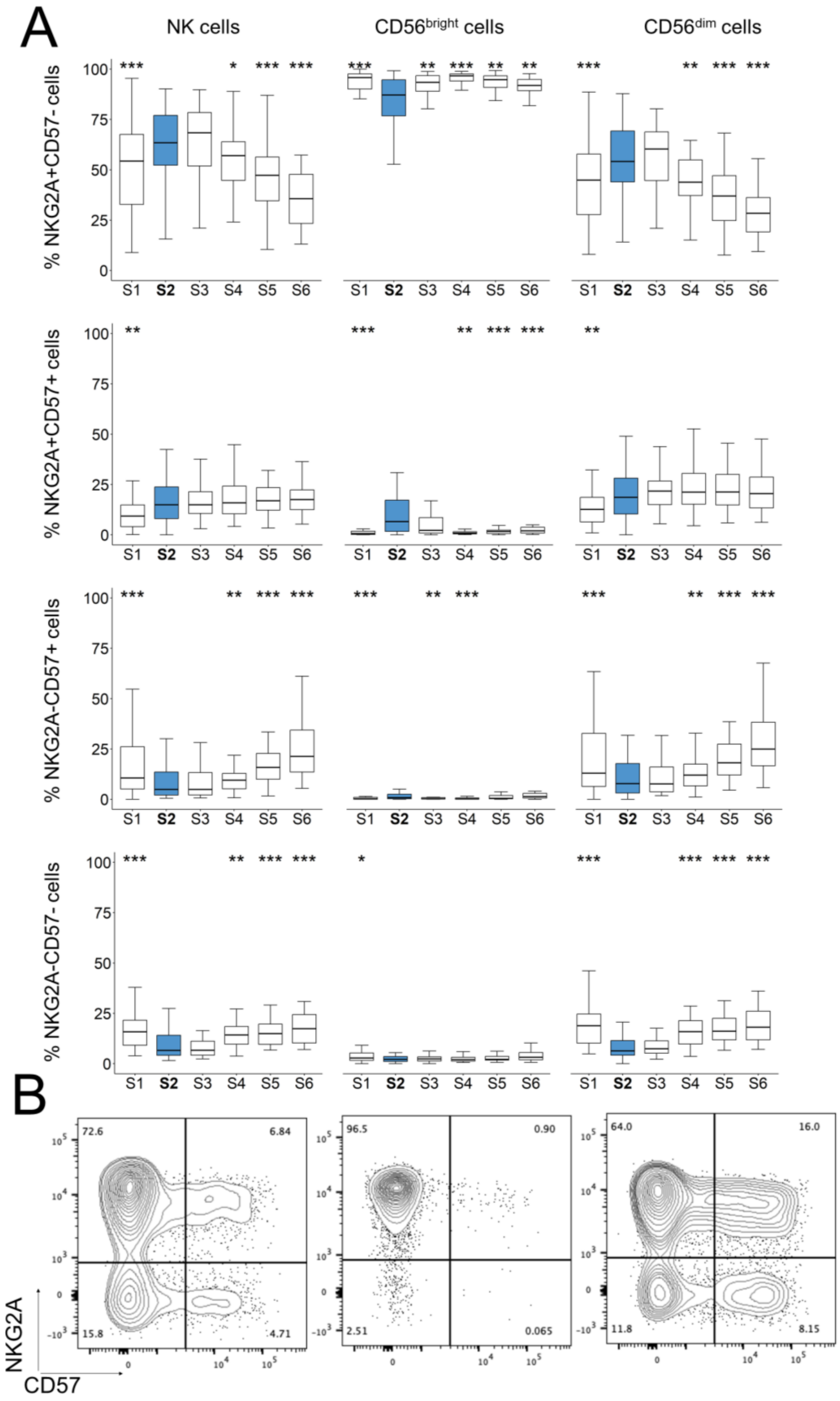

Figure S2. NK cell maturation status after autoHSCT. (A) Boxplots showing the percentage of NKG2A+CD57-, NKG2A+CD57+, NKG2A-CD57+ and NKG2A-CD57- cells within total NK cells, as well as within CD56^{bright} and CD56^{dim} NK cell subsets, at six time points (S1-S6). Boxplots display the median, interquartile range (IQR; 25–75th

percentiles), with whiskers indicating the minimum and maximum values. Statistical significance was determined by comparing each sample to S2 using the Wilcoxon matched-pairs signed-rank test: $*p < 0.05$, $**p < 0.01$, $***p < 0.001$; non-significant comparisons are not indicated. **(B)** Representative contour plots showing the three analyzed populations.

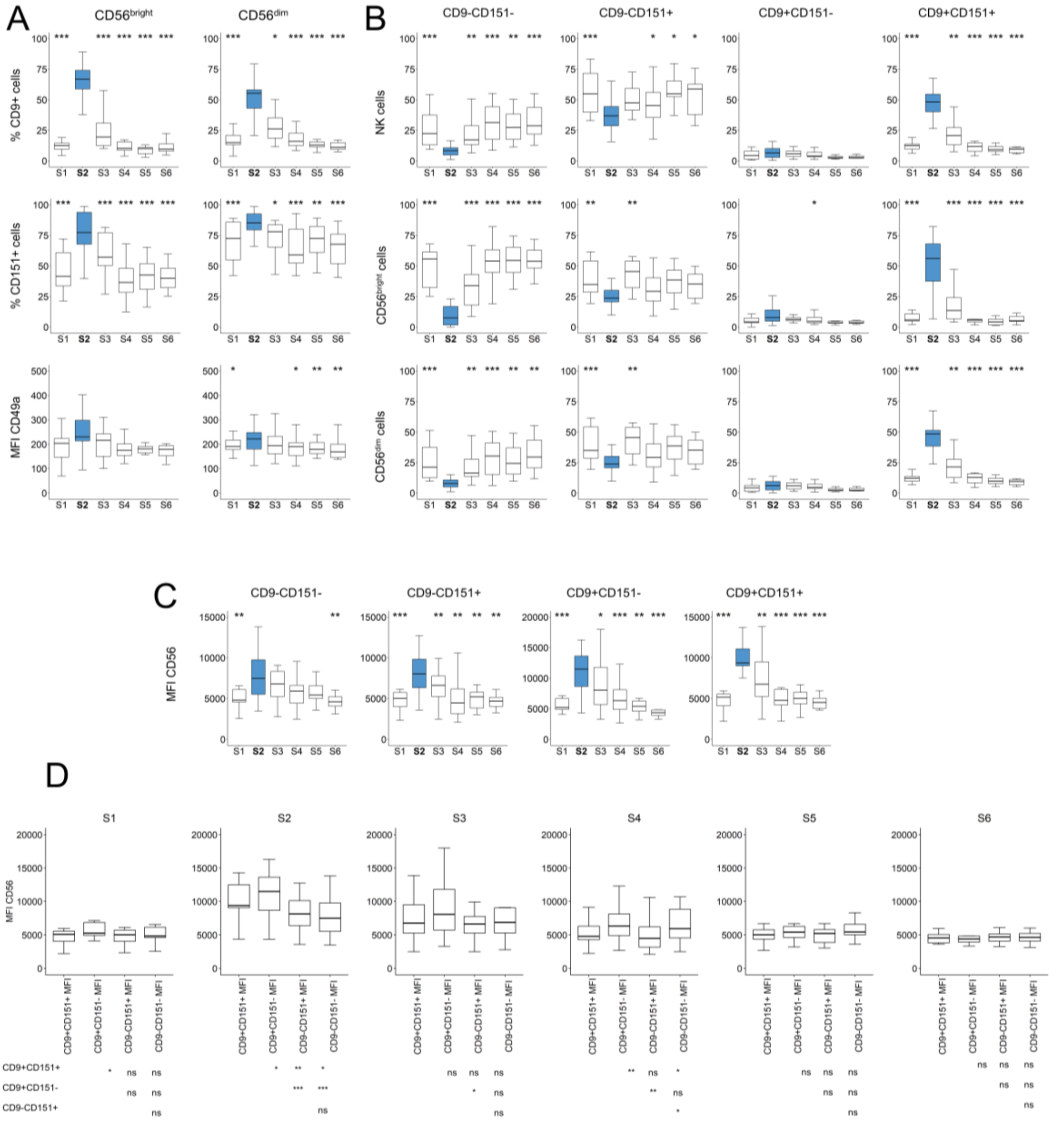

Figure S3. Transient acquisition of a decidual-like phenotype by NK cells early after autoHSCT. Boxplots showing the expression of decidual markers CD9, CD151 and CD49a in total NK cells and in CD56^{dim} and CD56^{bright} NK cell subsets at the six studied time points (S1-S6). **(A)** Percentage of cells expressing CD9 and CD151, and median fluorescence (MFI) of CD49a in CD56^{dim} and CD56^{bright} NK cell subsets. **(B)** Frequencies of CD9-CD151-, CD9-CD151+, CD9+CD151- and CD9+CD151+ cells within total NK cells, as well as in CD56^{dim} and CD56^{bright} NK cell subsets. **(C)** MFI of CD56 in CD9-CD151-, CD9-CD151+, CD9+CD151- and CD9+CD151+ in NK cells. Boxplots depict the median, IQR (25–75th percentiles), with whiskers indicating the minimum and maximum values. Statistical significance was determined by comparing each sample with S2 using Wilcoxon matched-pairs signed-rank test (*p < 0.05, **p < 0.01, ***p < 0.001; non-significant comparisons are not indicated). **(D)** MFI of CD56 across the six studied time points (S1-S6), comparing expression within CD9-CD151-, CD9-CD151+, CD9+CD151-, and CD9+CD151+ NK cells. Data are represented as boxplots with the median, IQR (25–75th percentiles), with whiskers denoting the lowest and highest values. Statistical comparisons between the four populations defined by CD9 and CD151

expression were performed using Wilcoxon matched-pairs signed-rank test (* $p < 0.05$, ** $p < 0.01$, *** $p < 0.001$; ns = not significant).

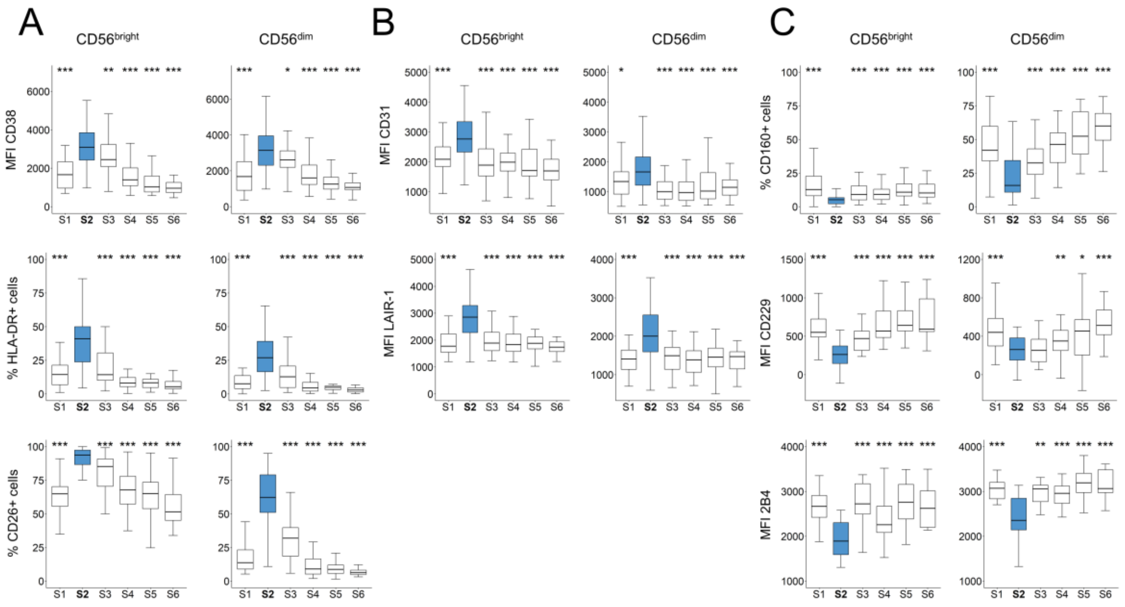

Figure S4. NK cell activation markers and expression of inhibitory and activating receptors after autoHSCT. Boxplots illustrating the expression of activation markers, inhibitory receptors, and activating receptors within CD56^{dim} and CD56^{bright} NK cell subsets across the six studied time points (S1-S6). **(A)** MFI of CD38 expression and the percentage of cells expressing HLA-DR and CD26. **(B)** MFI of CD31 and LAIR-1 expression. **(C)** Percentage of CD160+ cells and MFI of CD229 and 2B4 expression. Data are represented as boxplots with the median, IQR (25–75th percentiles), with whiskers denoting the lowest and highest values. Statistical significance was determined by comparing each sample to S2 using the Wilcoxon matched-pairs signed-rank test: *p < 0.05, **p < 0.01, ***p < 0.001; non-significant comparisons are not indicated.

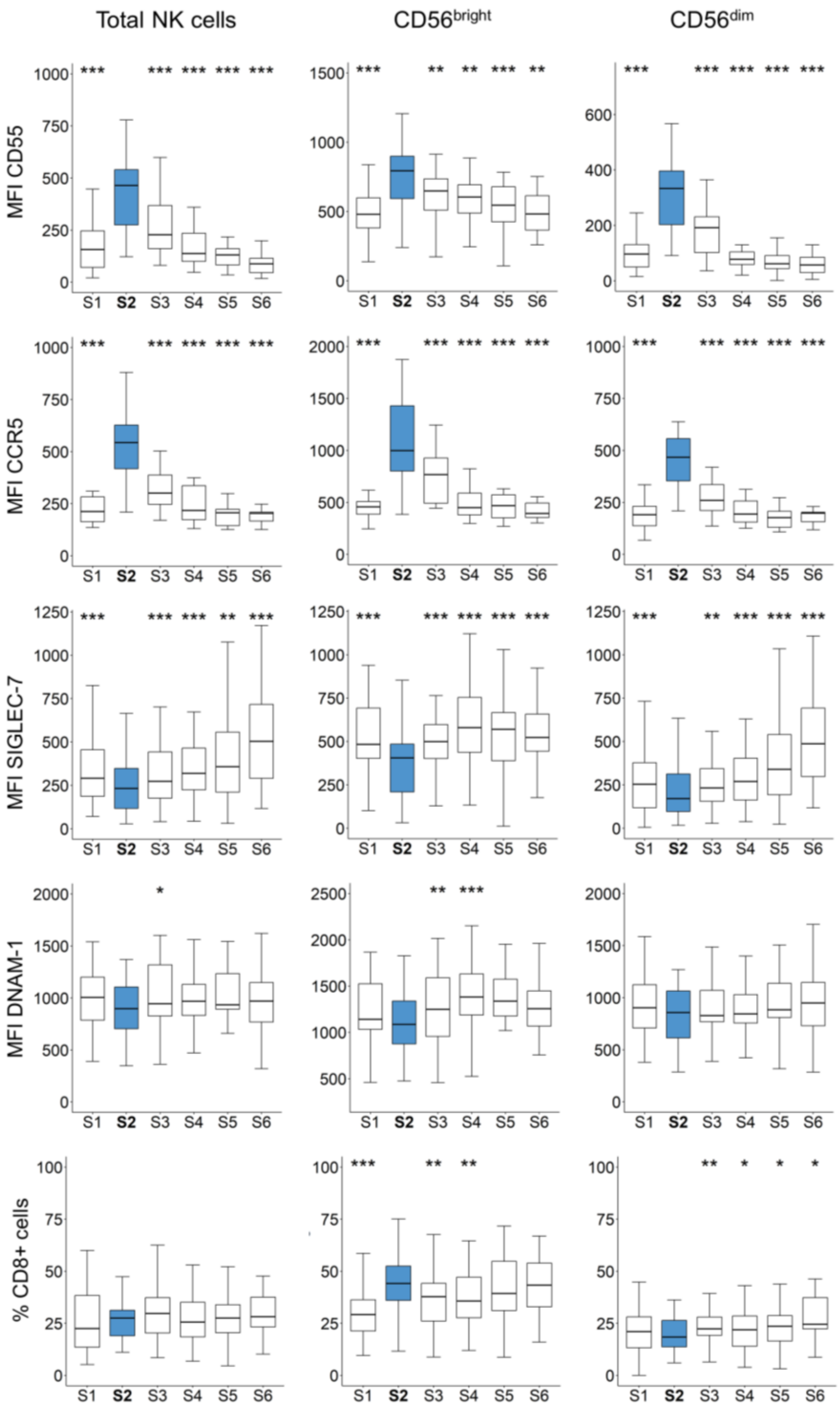

Figure S5. Additional alterations in NK cell receptors expression following autoHSCT. Boxplots depicting the expression of selected receptors in total NK cells and in CD56^{dim} and CD56^{bright} NK cell subsets across the six studied time points (S1-S6). **(A)** MFI of CD55, CCR5, SIGLEC-7, and DNAM-1 expression, as well as the percentage of CD8+ cells. Data are represented as the median and 25–75th percentiles, with whiskers denoting the lowest and highest values. Statistical significance was determined by comparing each sample to S2 using the Wilcoxon matched-pairs signed-rank test: *$p < 0.05$, **$p < 0.01$, ***$p < 0.001$; non-significant comparisons are not indicated.

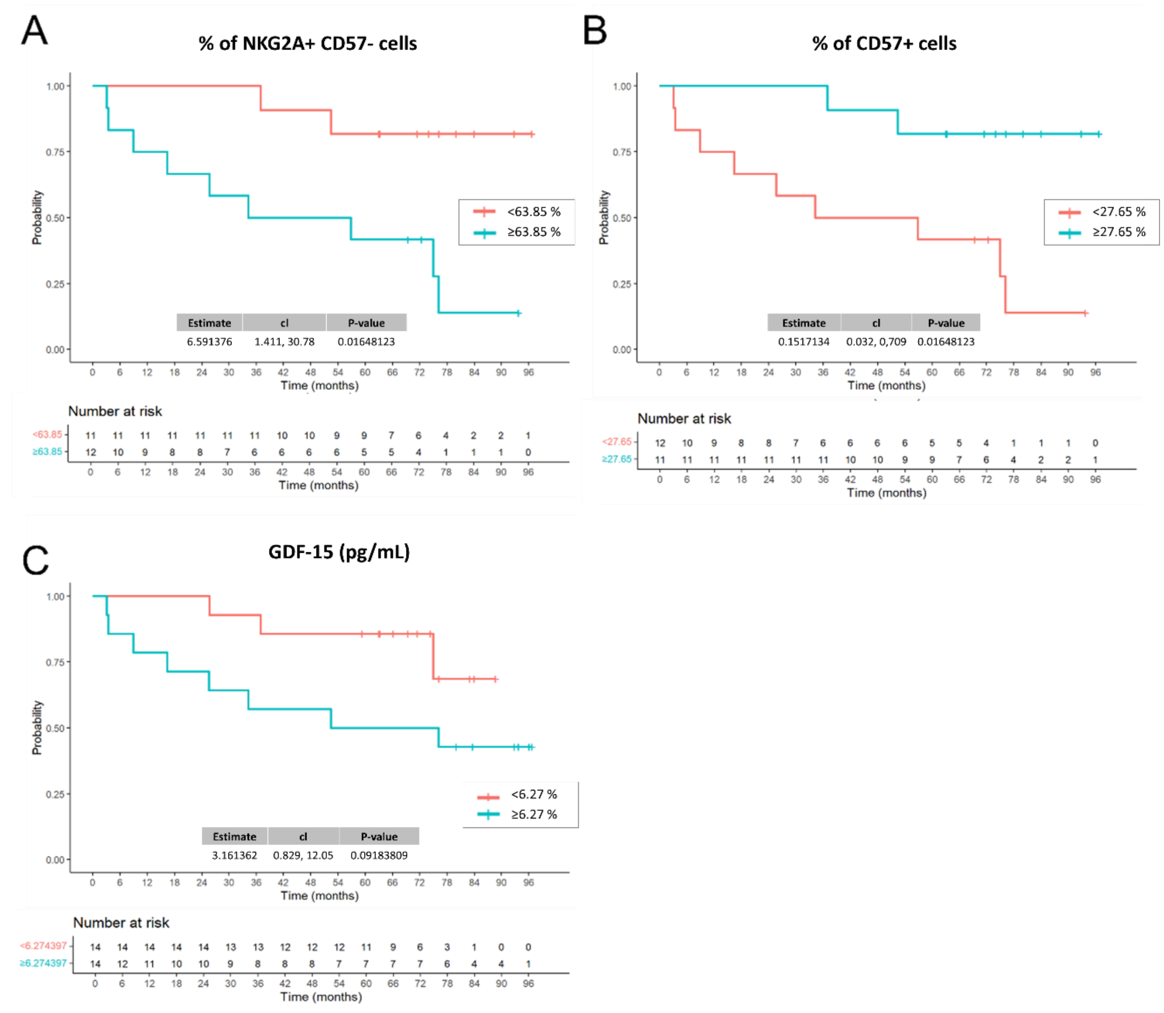

Figure S6. Altered NK cell phenotype and its potential impact on patient prognosis. Survival rates using Kaplan-Meier analysis of progression-free survival (PFS) in non-Hodgkin lymphoma patients undergoing autoHSCT. Patients were stratified based on the median frequencies of NKG2A+CD57− NK cells at S3 **(A)**, CD57+ NK cells at S3 **(B)**, and GDF-15 plasma levels at S2 **(C).** The hazard ratio estimate (estimate), lower and upper bounds of a 95% confidence interval (ci) and p-values are specified.

Table S1. Flow cytometry panels

Panel 1

Laser	Filter	Fluorochrome	Marker	Manufacturer	Clone
405	450/50	BV421	CD56	BD Biosciences	NCAM 16.2
	525/50	BV510	CD3/CD14/CD19/ CD123	BD Biosciences	UCHT1/MφP9/ SJ25C1/9F5
	525/50	Aqua Dead	Viability	Invitrogen	
	610/20	BV605	CD31	BD Biosciences	WM59
	670/30	BV650	KIR3DL1	BD Biosciences	DX9
	710/50	BV711	CD103	BioLegend	Ber-ACT8
	780/60	BV786	CD57	BioLegend	QA17A04
488	530/30	AF488	KIR2DS4	Miltenyi Biotec	REA860
	575/25	PE	KIR2DL2/L3/S2	Beckman Coulter	GL183
	610/20	PE-Dazzle594	CD9	BioLegend	HI9a
	710/50				
	780/60	PE-Vio770	NKp80	Miltenyi Biotec	4A4.D10
637	670/30	APC	NKG2A	Beckman Coulter	Z199
	730/45	AF700	Siglec-7	BioLegend	6-434
	780/60	APC-Vio770	KIR2DL1	Miltenyi Biotec	REA284

Panel 2

Laser	Filter	Fluorochrome	Marker	Manufacturer	Clone
405	450/50	BV421	CD56	BD Biosciences	NCAM 16.2
	525/50	BV510	CD3/CD14/CD19/ CD123	BD Biosciences	UCHT1/MφP9/ SJ25C1/9F5
	525/50	Aqua Dead	Viability	Invitrogen	
	610/20	BV605	HLA-DR	BD Biosciences	G46-6
	670/30	BV650	CD8	BD Biosciences	RPA-T8
	710/50	BV711	CD226 (DNAM-1)	BD Biosciences	DX11
	780/60	BV786	CD38	BD Biosciences	HIT2
488	530/30	AF488	CD160	BD Biosciences	BY55
	575/25	PE	NKG2C	R&D systems	134591
	610/20	PE-CF594	CD26	BD Biosciences	M-A261
	710/50	PerCP-Cy5.5	LAIR-1	BD Biosciences	DX26
	780/60	PE-Vio770	NKp80	Miltenyi Biotec	4A4.D10
637	670/30	APC	CD229	Miltenyi Biotec	Hly9.1.25
	730/45	AF700	CD55	R&D systems	278810
	780/60	APC-Cy7	CD57	Miltenyi Biotec	REA769

Panel 3

Laser	Filter	Fluorochrome	Marker	Manufacturer	Clone
405	450/50	BV421	CD56	BD Biosciences	NCAM 16.2
	525/50	BV510	CD3/CD14/CD19/ CD123	BD Biosciences	UCHT1/MφP9/ SJ25C1/9F5
	525/50	Aqua Dead	Viability	Invitrogen	
	610/20				
	670/30	BV650	CCR5	BD Biosciences	2D7/CCR5
	710/50				
	780/60				
488	530/30	FITC	2B4	BioLegend	C1.7
	575/25	PE	CD151	BD Biosciences	14A2.H1
	610/20	PE-Dazzle594	CD9	BioLegend	HI9a
	710/50	PerCP-Cy5.5	CD49a	BioLegend	TS2/7
	780/60	PE-Vio770	NKp80	Miltenyi Biotec	4A4.D10
637	670/30				
	730/45				
	780/60				

Panel 4

Laser	Filter	Fluorochrome	Marker	Manufacturer	Clone
405	450/50	BV421	CD56	BD Biosciences	NCAM 16.2
	525/50	BV510	CD3/CD14/CD19/ CD123	BD Biosciences	UCHT1/MφP9/ SJ25C1/9F5
	610/20	BV605	HLA-DR	BD Biosciences	G46-6
	670/30				
	710/50				
	780/60				
488	530/30	FITC	CD107a	Miltenyi Biotec	REA792
	575/25	PE	CD151	BD Biosciences	14A2.H1
	610/20	PE-Dazzle594	CD9	BioLegend	HI9a
	710/50				
	780/60				
637	670/30	APC	CD160	BD Biosciences	BY55
	730/45	R718	CD26	BD Biosciences	M-A261
	780/60	Near IR Dead	Viability	Invitrogen	

Table S2. Patients´ KIR haplotypes

	2DL1	2DL2	2DL3	2DL4	2DL5	3DL1	3DL2	3DL3	3DS1	2DS1	2DS2	2DS3	2DS4 variant	2DS5	2DP1	3DP1					
1	+	+	-	+	+	+	+	+	+	+	+	+	Del	+	+	+	CENB1	CENB1 or B2	TELA	TELB1	Bx73
2	+	+	+	+	+	+	+	+	+	+	+	-	Del	+	+	+	CENA	CENB2	TELA	TELB1	Bx3
3	+	-	+	+	-	+	+	+	-	-	-	-	Del	-	+	+	CENA	CENA	TELA	TELA	AA1
4	+	+	+	+	+	+	+	+	+	+	+	+	Del	+	+	+	CENA	CENB1	TELA	TELB1	Bx6
5	-	+	-	+	-	+	+	+	-	-	+	-	Full	-	-	+	CENB2	CENB2	TELA	TELA	Bx72
6	+	-	+	+	+	+	+	+	+	+	-	-	Full	+	+	+	CENA	CENA	TELA	TELB1	Bx2
7	+	+	+	+	+	+	+	+	+	+	+	+	Del	-	+	+	CENA	CENB1 or B2	TELA	TELB2	Bx7
8	+	-	+	+	-	+	+	+	-	-	-	-	Full, Del	-	+	+	CENA	CENA	TELA	TELA	AA1
9	+	-	+	+	-	+	+	+	-	-	-	-	Del	-	+	+	CENA	CENA	TELA	TELA	AA1
10	+	+	+	+	-	+	+	+	-	-	+	-	Full, Del	-	+	+	CENA	CENB2	TELA	TELA	Bx4
11	+	+	+	+	-	+	+	+	-	-	+	-	Full, Del	-	+	+	CENA	CENB2	TELA	TELA	Bx4
12	+	-	+	+	+	+	+	+	+	+	-	-	Del	+	+	+	CENA	CENA	TELA	TELB1	Bx2
13	+	+	+	+	+	+	+	+	-	-	+	+	Full, Del	-	+	+	CENA	CENB1	TELA	TELA	Bx5
14	+	+	+	+	+	+	+	+	+	+	+	+	Del	-	+	+	CENA	CENB1 or B2	TELA	TELB2	Bx7
15	+	+	+	+	+	+	+	+	+	+	+	-	Full	+	+	+	CENA	CENB2	TELA	TELB1	Bx3
16	-	+	-	+	-	+	+	+	-	-	+	-	Full, Del	-	-	+	CENB2	CENB2	TELA	TELA	Bx72
17	+	+	-	+	+	+	+	+	-	-	+	+	Del	-	+	+	CENB1	CENB1 or B2	TELA	TELA	Bx71
18	+	+	+	+	-	+	+	+	-	-	+	-	Del	-	+	+	CENA	CENB2	TELA	TELA	Bx4
19	+	+	-	+	+	+	+	+	-	-	+	+	Full, Del	-	+	+	CENB1	CENB1 or B2	TELA	TELA	Bx71
20	+	-	+	+	-	+	+	+	-	-	-	-	Del	-	+	+	CENA	CENA	TELA	TELA	AA1
21	+	-	+	+	-	+	+	+	-	-	-	-	Del	-	+	+	CENA	CENA	TELA	TELA	AA1
22	+	-	+	+	-	+	+	+	-	-	-	-	Full, Del	-	+	+	CENA	CENA	TELA	TELA	AA1
23	+	+	+	+	-	+	+	+	-	-	+	-	Full, Del	-	+	+	CENA	CENB2	TELA	TELA	Bx4
24	+	+	+	+	-	+	+	+	-	-	+	-	Del	-	+	+	CENA	CENB2	TELA	TELA	Bx4
25	+	+	+	+	-	+	+	+	-	-	+	-	Del	-	+	+	CENA	CENB2	TELA	TELA	Bx4
26	+	-	+	+	+	+	+	+	+	+	-	-	Full	+	+	+	CENA	CENA	TELA	TELB1	Bx2
27	+	+	-	+	+	+	+	+	+	+	+	+	Del	+	+	+	CENB1	CENB1 or B2	TELA	TELB1	Bx73
28	+	-	+	+	+	-	+	+	+	+	-	+	-	+	+	+	CENA	CENA	TELB1	TELB2	Bx75
29	+	+	+	+	-	+	+	+	-	-	+	-	Del	-	+	+	CENA	CENB2	TELA	TELA	Bx4
30	+	+	+	+	+	+	+	+	-	-	+	+	Del	-	+	+	CENA	CENB1	TELA	TELA	Bx5
31	+	-	+	+	-	+	+	+	-	-	-	-	Full	-	+	+	CENA	CENA	TELA	TELA	AA1
32	+	+	+	+	+	+	+	+	+	+	+	+	Full	-	+	+	CENA	CENB1 or B2	TELA	TELB2	Bx7
33	+	+	+	+	+	+	+	+	+	-	+	+	Full	-	+	+	CENA	CENB2	TELA	TELB2*	Bx13
34	+	+	+	+	-	+	+	+	-	-	+	-	Del	-	+	+	CENA	CENB2	TELA	TELA	Bx4
35	+	-	+	+	+	+	+	+	+	+	-	+	Full	-	+	+	CENA	CENA	TELA	TELB2	Bx8
36	+	+	+	+	-	+	+	+	-	-	+	-	Del	-	+	+	CENA	CENB2	TELA	TELA	Bx4
37	+	+	+	+	+	+	+	+	+	+	+	+	Full	-	+	+	CENA	CENB1 or B2	TELA	TELB2	Bx7
38	+	+	+	+	+	+	+	+	+	+	+	-	Del	+	+	+	CENA	CENB2	TELA	TELB1	Bx3
39	+	-	+	+	+	+	+	+	+	+	-	-	Del	+	+	+	CENA	CENA	TELA	TELB1	Bx2
40	+	+	+	+	-	+	+	+	-	-	+	-	Del	-	+	+	CENA	CENB2	TELA	TELA	Bx4
41	+	+	+	+	+	+	+	+	+	-	+	+	-	-	+	+	CENA	CENB2	TELA	TELB2*	Bx13
42	+	-	+	+	+	+	+	+	+	+	-	-	Del	+	+	+	CENA	CENA	TELA	TELB1	Bx2
43	+	-	+	+	+	-	+	+	+	+	-	+	-	+	+	+	CENA	CENA	TELB1	TELB2	Bx75
44	+	+	+	+	+	+	+	+	-	-	+	+	Del	-	+	+	CENA	CENB1	TELA	TELA	Bx5
45	+	-	+	+	+	+	+	+	+	+	-	-	Del	+	+	+	CENA	CENA	TELA	TELB1	Bx2
46	+	+	+	+	-	+	+	+	-	-	+	-	Del	-	+	+	CENA	CENB2	TELA	TELA	Bx4
47	+	+	+	+	+	+	+	+	-	-	+	+	Full, Del	-	+	+	CENA	CENB1	TELA	TELA	Bx5
48	+	-	+	+	+	+	+	+	+	+	-	-	Del	+	+	+	CENA	CENA	TELA	TELB1	Bx2
49	+	-	+	+	-	+	+	+	-	-	-	-	Full	-	+	+	CENA	CENA	TELA	TELA	AA1

Genes are included (+) or excluded (-). For 2DS4, gene variants were studied in both alleles as full gene (Full) or a deletion variant (Del).
